# Trou maculaire contusif

**DOI:** 10.11604/pamj.2014.17.213.4052

**Published:** 2014-03-18

**Authors:** Ryme Abdelkhalek, Mehdi Khmamouch

**Affiliations:** 1 Service d'Ophtalmologie, Hôpital Militaire d'Instruction Mohamed V, Rabat, Maroc

**Keywords:** macular hole, contusion, eye, Optical Coherence Tomography, Trou maculaire, contusion, œil, Tomographie en Cohérence Optique

## Image en medicine

Il s'agit d'un enfant âgé de 10 ans, victime d'un traumatisme post contusif au niveau de l'oeil droit. L'examen de l'oeil droit trouve une acuité visuelle aux comptés des doigts. Le reflexe photo moteur est paresseux. Le fond d'oeil montre une hémorragie fovéolaire avec oedème périfovéolaire. Hémorragie rétinienne au-dessous de la macula. La Tomographie en Cohérence Optique (OCT) montre un trou maculaire de pleine épaisseur mesurant 451µm. Dans un premier temps, une surveillance a été indiquée, vu la possibilité d'une fermeture spontanée du trou avec la prescription d'une corticothérapie par voie orale pendant cinq jours.

**Figure 1:**
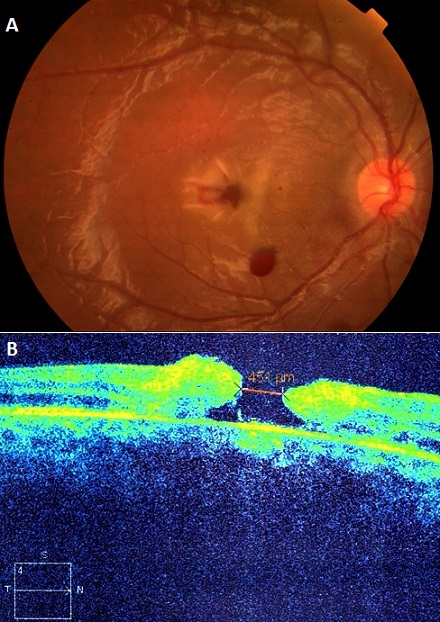
A) FO, Fond d'œil montrant une hémorragie fovéolaire avec œdème péri-maculaire; B)OCT montrant trou maculaire de pleine épaisseur mesurant 451µm

